# Discovery of three loci increasing resistance to charcoal rot caused by *Macrophomina phaseolina* in octoploid strawberry

**DOI:** 10.1093/g3journal/jkab037

**Published:** 2021-03-16

**Authors:** Jonathan R Nelson, Sujeet Verma, Nahla V Bassil, Chad E Finn, James F Hancock, Glenn S Cole, Steven J Knapp, Vance M Whitaker

**Affiliations:** 1 Department of Horticultural Science, University of Florida, IFAS Gulf Coast Research and Education Center, 14625 CR 672 Wimauma, FL 33598, USA; 2 USDA-ARS National Clonal Germplasm Repository, Corvallis, OR 97333, USA; 3 USDA-ARS, HCRU, 3420 NW Orchard Avenue, Corvallis, OR 97330, USA (posthumous); 4 Department of Horticulture, Michigan State University, East Lansing, MI 48824, USA; 5 Department of Plant Sciences, University of California, Davis, CA 95616, USA

**Keywords:** fruit breeding, GWAS, haplotype, multiparental population, pedigree-based analysis, QTL

## Abstract

Charcoal rot caused by *Macrophomina phaseolina*is an increasing economic problem in annualized strawberry production systems around the world. Currently there are no effective postfumigation chemical controls for managing charcoal rot, and no information is available on the genetic architecture of resistance to *M. phaseolina* in strawberry (*Fragaria ×ananassa*). In this study, three multiparental discovery populations and two validation populations were inoculated at planting and evaluated for mortality in three consecutive growing seasons. Genome-wide SNP genotyping and pedigree-based analysis with FlexQTL™ software were performed. Two large-effect quantitative trait loci (QTL) increasing charcoal rot resistance were discovered and validated in cultivated germplasm. *FaRMp1* was located on linkage group 2A in the interval 20.4to 24.9 cM, while *FaRMp2* was located on linkage group 4B in the interval 41.1to 61.2 cM. Together these QTLs explained 27% and 17% of the phenotypic variance in two discovery populations consisting of elite breeding germplasm. For both QTLs, the resistant allele showed some evidence of partial dominance, but no significant interaction was detected between the two loci. As the dosage of resistant alleles increased from 0 to 4 across the two QTLs, mortality decreased regardless of the combination of alleles.A third locus, *FaRMp3* on 4D, was discovered in FVC 11–58, a reconstituted *F.×ananassa* originating from diverse *F. virginiana* and *F. chiloensis* accessions. This locus accounted for 44% of phenotypic variation in four segregating crosses. These findings will form the basis for DNA-informed breeding for resistance to charcoal rot in cultivated strawberry.

**KEY MESSAGE**

Charcoal rot of strawberry, caused by *Macrophomina phaseolina*, is an increasing economic problem in strawberry production worldwide, with limited means of control besides genetic resistance. Three different genetic loci for resistance were discovered, each significantly slowing the rate of mortality. Together these loci will form a basis for DNA-informed breeding tools against this disease.

## Introduction

The cultivated allo-octoploid strawberry *Fragaria*×*ananassa* (2*n *=* *8*x *=* *56) is one of the most economically valuable crops within the Rosaceae. Strawberry fruit are rich in health-associated compounds including vitamins C and K, fiber, folic acid, manganese, and potassium (CSC 2015; Lewin 2016). Strawberry is a hybrid species originating from an accidental cross in Europe between the two wild octoploids, *F. chiloensis* from Chile and *F. virginiana* from eastern North America in the mid-18th century (Darrow 1966). The first chromosome-scale genome assembly of cultivated strawberry recently shed light on the most ancient origins of the species, revealing four diploid progenitors (Edger *et al.* 2019).

Charcoal rot, caused by the soilborne fungus *M. phaseolina*, causes rapid collapse and death of strawberry plants. Infection by *M. phaseolina* is favored by high temperatures (∼30°C), low-soil moisture, and sandy soils (Peres *et al.* 2018). This describes typical strawberry growing conditions in California, Florida, and many other strawberry-producing regions around the globe. The fumigant methyl bromide was previously the industry standard for controlling soilborne pathogens in strawberry production, but with its progressive phase-out (Montzka *et al.* 2002; Velders *et al.* 2007, Holmes *et al.* 2020) charcoal rot has rapidly become a major disease of strawberry. Alternative fumigants have limited movement through the soil profile leading to decreased effectiveness, and there are no curative fungicides for this disease (Peres *et al.* 2018).

Genetic resistance holds promise as a method of control. Differences in resistance in elite germplasm and released cultivars have recently been documented (Holmes *et al.* 2020). Resistance has also been documented in *F. chiloensis* and *F. virginiana* and in reconstituted *F.*×*ananassa* (Zurn *et al.* 2020) generated from diverse sources of both species (Hancock *et al.* 2010). Even moderate reductions in the rate of progression and severity of charcoal rot can be economically beneficial, as a strawberry plant that survives longer will produce more marketable yield. At present, however, there is no information available on the genetic architecture of resistance to charcoal rot in strawberry. Thus, there is a critical need for research on genetic resistance to *M. phaseolina* in strawberry and for development of tools for DNA-informed breeding for resistance.

Most fruit breeding programs are characterized by collections of pedigree-connected clones, complex multigenerational families with variable numbers of progeny, and diverse genetic backgrounds (Bink *et al.* 2002, 2014). To detect loci and their effects across representative genetic backgrounds, it is advantageous to simultaneously analyze pedigree-connected families that represent the full genetic variability of a population (Mangandi *et al.* 2017). Pedigree-based quantitative trait loci(QTL) analysis using FlexQTL™ software (Bink *et al.* 2014) and genome-wide association study (GWAS) approaches (Wang and Zhang 2020) can both be applied to detect loci in complex breeding populations. FlexQTL™ utilizes Bayesian analysis methods with Markov chain Monte Carlo (MCMC) algorithms to estimate chromosomal location, number, mode, and magnitude of QTL in unbalanced population sets. It also provides the advantage of marker haplotype phasing based on recombination events and can trace resistant alleles back to founders using identity-by-descent. In thisstudy, we utilize both FlexQTL™ and GWAS to characterize QTL and their effects in breeding germplasm.

The primary objectives of this study were to: (1) discover and validate genetic loci increasing resistance to *M. phaseolina* in *F.*×*ananassa* and (2) characterize the effects and interactions of QTL alleles in breeding germplasm. To achieve these objectives, multiparental populations segregating for resistance were developed by collaborating U.S. breeding programs and screened via inoculated field trials over multiple years at the University of Florida (UF).

## Materials and methods

### QTL discovery populations

Three multiparental discovery populations were inoculated, planted, and evaluated in three consecutive growing seasons in 2016–2017, 2017–2018, and 2018–2019. Populations in the first 2 years consisted of seedlings obtained from controlled crosses among elite lines within the UF strawberry breeding program. Crosses were chosen to represent the breadth of the elite germplasm as well as to achieve connectedness among parents (Supplementary Figures 1 and 2). A network analysis was performed to visually examine the relationships within discovery populations (Purcell *et al.* 2007; Neuditschko *et al.* 2012) (Supplementary Figure 3). The 2016–2017 population consisted of 40 full-sib families obtained from crosses made among 40 parents (Table 1). The 2017–2018 population consisted of 33 full-sib families derived from crosses among 29 parents (Table 1). Although no cross was evaluated across years, seven parents were common between the 2 years providing replication of segregating alleles across years. In 2018–2019, four crosses were evaluated that were derived from FVC 11–58, a reconstituted *F. ×ananassa* of diverse parentage that was previously identified as resistant to charcoal rot (Zurn *et al.* 2020). This selection was crossed to a single susceptible advanced selection each from the Michigan State University, UF, University of California-Davis and USDA-ARS (Corvallis, OR) breeding programs (Table 2). All seedlings, parents, and checks were clonally multiplied via runners in the UF summer breeding nursery near Malin, Oregon. Four clonal replicates (runners) of each seedling were planted in a randomized complete block field design (RCBD) with one runner plant in each of the four blocks (two raised-beds covered with black plastic mulch) at the UF/IFAS Gulf Coast Research and Education Center in Wimauma, Florida. Each plant was inoculated at the time of planting on October 6, 2016, October 6, 2017, and September 27, 2018 into raised beds covered with black plastic mulch. Plants were established with overhead irrigation for 10days after planting during daylight hours. Drip irrigation was used for the remainder of the growing season. Fertilizer applications and pest control were carried out according to industry standards.

**Table 1:** Summary statistics of QTL discovery and validation populations evaluated for resistance to charcoal rot caused by *Macrophomina phaseolina*. Parents and seedlings were clonally propagated via runners for field evaluations

	Discovery			Validation	
	2016–2017	2017–2018	2018–2019	2017–2018	2018–2019
Parents	40	29	5		
Full-sib families	40	33	4	–	–
Individuals/family	10–26	10–50	16–55	–	–
Clones/individual	2–4	4	4	8	8
Check cultivars	35	42	–	–	–
2016 seedlings	–	–	–	79	
2017 seedlings	–	–	–	–	37
2018 seedlings	–	–	–	–	84
Elite selections	–	–	–	13	13
Commercial cultivars	–	–	–	4	5
Total individuals	576	579	128	96	149
Total plants evaluated	2,414	2,646	512	768	1192

**Table 2:** Crosses for QTL discovery in 2018–2019 derived from reconstituted *F.*×*ananassa* FVC 11–58 (resistance source)

Family	Mother	Father	*n*
18.41	FL 13.42-5	FVC 11–58	55
18.68	05C109P002	FVC 11–58	16
ORUS 3792	FVC 11–58	ORUS 2780-1	31
ORUS 3801	MSU61	FVC 11–58	26

### QTL validation populations

QTL validation populations were evaluated over two growing seasons.The 2017–2018 population was planted on October 6, 2017 and consisted of 96 individuals including 79 selections, 13 elite selections, and 4 commercial varieties. The 2018–2019 population was planted on October 10, 2018 and consisted of 149 individuals including 121 selections, 13 elite selections, and 5 commercial cultivars. Eight clonal replicates were tested, split into two plots of four plants each. The trials were planted as an RCBD, where each block consisted of one raised bed. Validation sets were planted adjacent to the discovery populations tested in the same years and inoculated according to the same protocol.

### Inoculation

*M. phaseolina* inoculum was produced by reviving three isolates from −80°C storage on September 16, 2016, September 22, 2017, and September 5, 2018. Isolates 01-179, 02-200, and 09-95 were used in year 1, isolates 09-95, 12-467, and 17-29 were used in year 2, and isolates 09-95, 12-467, and 17-29 were used in year 3. Each isolate had been previously collected from strawberry fruiting fields in central Florida by the GCREC small fruit pathology lab. These isolates were chosen as a representative sample of all isolates collected from strawberry in the region with at least an average level of pathogenicity. Isolates were revived on potato dextrose agar and incubated in the dark at 29°C for 3weeks. For every 10colonized plates, 400 mL deionized H_2_O was added and the mixture blended at low speed for 1minute. This solution was further diluted by adding 1,500 mL of sterilized 0.35% water agar solution in 2016 and 0.25% solution in 2017 and 2018. The total volumes of inoculum were 34, 48, and 32 liters in years 1, 2, and 3, respectively. This allowed for 8 liters of inoculum per replicate or 4 liters per bed. Concentrations of the final suspensions were 2.8 × 10^6^ microsclerotia/L in 2016, 2.7 × 10^6^ microsclerotia/L in 2017, and 3.12 × 10^3^ microsclerotia/L in 2018. The roots of each bare root transplant were dipped into the suspension up to the base of the crown for 3–5 seconds just prior to planting.

### Disease ratings

Ratings for the discovery populations began with the collapse of the first plants in the trial on October 28, 2016, November 1, 2017, and October 22, 2018 and continued until 75% of the entire plant population collapsed. Plant collapse was recorded weekly with a 0 assigned to surviving plants, and a 1 assigned to collapsed plants. Plants were considered collapsed when 75% or more of the total leaf canopy, considering leaves from all developed crowns, of each plant had wilted and collapsed onto the bed surface. Re-isolations from necrotic portions of crowns of randomly chosen collapsed plants were performed multiple times each season and confirmed *M. phaseolina* infection. Collapse over time was used to calculate the area under the disease progress curve (AUDPC) for each individual. Rating of the validation population in 2017–2018 began on November 8, 2017 and concluded on March 14, 2018 when mortality reached 66%. Rating of the validation set in 2018–2019 began on October 29, 2018 and concluded on January 9, 2019 when plant collapse reached 46%, since after that date mortality from other pathogens began to be detected in re-isolations. A single AUDPC value per individual was generated for QTL analysis by considering mortality over time of the multiple clonal replicates for each individual.

### Genotyping

DNA extractions for all populations were carried out according to Mangandi *et al.* (2017). Genotyping was performed using the Affymetrix Axiom^®^ IStraw35 SNP array (Verma *et al.* 2017) in 2016–2017 and 2018–2019 and the FanaSNP 50 K array (Hardigan *et al.* 2020) in 2018–2019. Genotyping errors were detected and removed by comparing SNP diplotypes of each individual to their respective parental genotypes and siblings. Correct parentage was inferred where incorrect parentage was detected. If the correct parent was not obvious, a “dummy” parent was assigned. In addition, markers with frequent inheritance errors, particularly on linkage groups (LGs) 2A and 4B, were removed from the dataset. These errors were determined in the “mconsistency” and “genomeIM” files of FlexQTL™ outputs and re-analyzed in FlexQTL™ multiple times until no errors were observed.

### Genetic mapping

An in-house genetic map “14.95” (Verma *et al.* unpublished) was used to assign SNP markers to 28 LGs of octoploid strawberry. The 14.95 map was constructed from 165 progeny derived from a cross between “FL 08-10” and “FL 12.115-10” using JoinMap 4.1 software (Van Ooijen 2006, 2011). The marker order and subgenome assignments followed the SSR reference map of Van Dijk *et al.* (2014). Fraction values of observed (oDR) and expected (eDR) double recombinant singletons were identified using FlexQTL™ software and markers with oDR—eDR ≥ 0.05 were eliminated from the map. A total of 14,332 SNP marker loci were genetically mapped. To reduce computation time in FlexQTL™, marker density was decreased by eliminating markers at identical map positions with the lowest minor allele frequency (MAF). A small percentage of markers with MAF below 0.1 were retained to capture unique map positions. To further curate the map, graphical genotyping was performed following the procedure of Bassil *et al.* (2015) for the LGs that showed a QTL. A final set of 9,670 markers that were highly informative for both the 2016–2017 and 2017–2018 discovery populations was used for QTL analysis. For 2018–2019 discovery population, ∼6,000 common SNP markers between IStraw35 and FanaSNP arrays were scrutinized. Of these, 3,402 SNPs were already included in the 14.95 genetic map and were used for the QTL analysis.

### QTL analysis and GWAS

QTL analysis was performed using FlexQTL™, which simulates MCMC-based Bayesian analysis using an additive model and incorporated the infinitesimal model (TIM) to account for polygenic effects, detailed in Bink *et al.* (2014). The maximum number of QTL was set to 15, the prior number of QTL was set to either 2 or 3 (Bink *et al.* 2002, 2014), and genome-wide analyses were performed three times for each prior. To save computational time, the final map was further curated by keeping every fourth marker, equating to 25% of mapped markers, with the exception of the two LGswhere QTL were initially located. In all, there were 2,608 markers used in each iteration of the genome-wide QTL analyses. Following the genome-wide analyses, three iterations were run with only the LGswhere the QTL were detected (2A and 4B) for comparison. For all analyses, different starting seeds were used in order to create independence between runs, using simulation chain lengths of 100,000, 150,000, and 200,000 iterations with thinning values of 100, 150, and 200 respectively. The effective sample size was set to 101 to ensure convergence with effective chain sizes of at least 100. Each iteration converged with effective chain samples or ECS ≥ 100 for each of the parameters—mean (mean1), variance of the error (vERR1 1), variance of the polygenic model (vTIM1 1), number of QTL (nQTL), and the variance for the number of QTL (vQTL1 1) as recommended by Bink *et al.* (2014). The Bayes factor parameter (2lnBF_10_) was interpreted as giving nonsignificant (0–2), positive (2–5), strong (5–10), or decisive (>10) evidence for presence of QTL (Kass and Raftery 1995). VisualFlexQTL™ (www.flexqtl.nl) software was used to visualize FlexQTL™ outputs including traces of convergence of QTL models, positions, and QTL genotypes. The mode value (identified in the Flex.log file) was used as the most probable location for each QTL. Complete FlexQTL™ parameters and their explanations are provided in Mangandi *et al.* (2017).

Becausethe markers on the FanaSNP array are anchored to the “Camarosa” genome (Edger *et al.* 2019; Hardigan *et al.* 2020), a GWAS of the 2018–2019 discovery population was performed using the physical positions of 49,331 SNPs using mixed linear models (MLMs) in the Genome Association and Prediction Integrated Tool in R (GAPIT version 3) (Wang and Zhang 2020). We did not perform SNP filtering prior to GWAS analysis and used default parameters for the MLM.

### Genetic parameters estimates

The phenotypic variance explained (PVE) by each QTL was estimated using outputs derived from FlexQTL™ software. Narrow-sense heritability (*h^2^*) was calculated using the formula:

$$h^{2}=\frac{VP-VE}{VP}$$

Where *VP* is the total phenotypic variance of the trait explained by all genetic factors included in the genetic model and *VE* is the residual error variance (Bink *et al.* 2014). The proportion of PVE by each QTL was calculated using the formula:

$$\mathrm{PVE}=\left(\frac{\mathrm{AVt}}{VP}\right)\times 100$$

where *AVt* is the additive variance of the trait being evaluated and *VP* is the total phenotypic variance of the trait as determined by the QTL model.

Becausethe phenotype inputs for FlexQTL™ analyses were a single AUDPC value per individual, generated from the collapse over time of the multiple clonal replicates of that individual, variability among replicates was not directly considered when calculating genetic parameters in FlexQTL™. To take individual replicates into account in calculating genetic parameters, variance components were calculated for years 1 and 2, first separately, then together, using ASReml (Gilmour *et al.* 2009). Incidence of plant collapse (0 or 1 for each plant) at the last evaluation date for each season was used. Univariate analyses for plant collapse were based on the binary scale (0 to 1) and analyzed using general linear mixed models (GLMMs). Variance components were estimated following Mangandi *et al.* (2017). All analyses were based on a parental model with a pedigree tracing back two generations. Replication was treated as a fixed effect, while bed, family, and genotype were considered as random effects. Broad sense (*H^2^*) and narrow sense (*h^2^*) heritability for each variable were estimated as follows:

$$H^{2}=\frac{\sigma_{G}^{2}}{{2\sigma }_{\mathrm{gca}}^{2}+\sigma_{\mathrm{sca}}^{2}+\sigma_{cl(f)}^{2}+\sigma_{e}^{2}}$$

 

$$h^{2}=\frac{4\sigma_{\mathrm{gca}}^{2}}{{2\sigma }_{\mathrm{gca}}^{2}+\sigma_{\mathrm{sca}}^{2}+\sigma_{cl(f)}^{2}+\sigma_{e}^{2}}$$

The variance components *σ^2^_G_, σ^2^_gca_, σ^2^_sca_, σ^2^_cl(f_*_)_, and *σ^2^_e_*were derived similarly to the methods of Osorio *et al.* (2014). The error variance (σ^2^_e_) of the estimates for plant collapse caused by *M. phaseolina* was fixed within GLMM to a value of 3.29 (Gilmour *et al.* 2009).

### Haplotyping and allele effects estimation

PediHaplotyper software (Voorrips *et al.* 2016) was utilized to identify haploblock alleles within two QTLs regions (Voorrips *et al.* 2016; Verma *et al.* 2019). Haplotypes were initially constructed for the 2016–2017 and 2017–2018 discovery populations using 28 SNP markers spanning 21to 24 cM on LG 2A and 16 SNP markers spanning 41to 50 cM on LG 4B. Two SNPs for each QTL, representing the full haplotype diversity in the populations with the fewest possible markers, were used for manual curation of the haplotypes (Table 3). Imputation of the missing marker information was carried out using the pedigree-based imputation method in *FImpute* software developed by Sargolzaei *et al.* (2014) (test dataset accuracy of 97%).

**Table 3:** Genetic parameter estimates for plant collapse caused by *Macrophomina phaseolina* for QTL discovery populations using both FlexQTL™ (calculated both genome-wide and considering linkage groups with significant QTL only) and ASReml software. *H^2^*=broad-senseheritability; *h^2^*=narrow-senseheritability; *d^2^*=dominancevariance;and *i^2^* =epistasicvariance.Standard errors are in parentheses. Phenotypic variance explained (PVE) by two QTL are also shown

	FlexQTL™					ASReml	
	Genome-wide			LGs 2A and4B			
	2016–2017	2017–2018	2018–2019	2016–2017	2017–2018	2016–2017	2017–2018
***H^2^***	–	–	–	–	–	0.46 (0.04)	0.31 (0.03)
***h^2^***	0.49–0.52	0.33–0.53	0.43–0.44	0.52	0.52–0.57	0.33 (0.10)	0.11 (0.09)
***d^2^***	–	–	–	–	–	0.00 (0.00)	0.19 (0.10)
***i^2^***	–	–	–	–	–	0.07 (0.07)	0.00 (0.08)
**PVE (%)**	20–36	13–17	79–82	20–34	13–21	–	–

Using one-way ANOVA in R software (R Core Team 2019), statistical significance of the phenotypic effects was determined for each haplotype on LGs 2A and 4B separately. The general linear hypothesis was adjusted for multiple comparisons using false discovery rate. Diplotype effects were evaluated using the same method except that the general linear hypothesis was adjusted using the Bonferroni test for multiple comparisons.Haplotypes and diplotypes were used to represent and characterize QTL allele and genotype effects, respectively.

## Results

**Figure 1:**
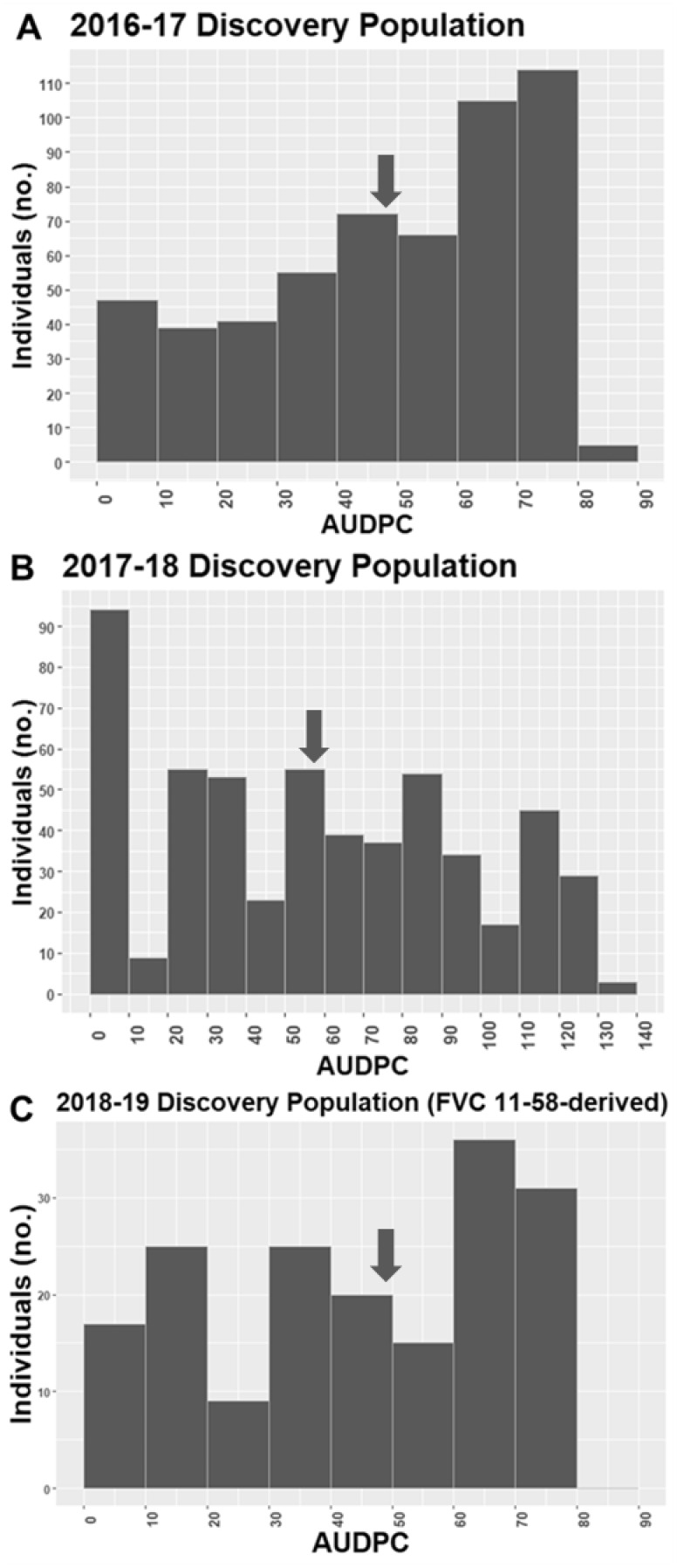
Frequency distributions of AUDPC values for QTL discovery populations tested over three field seasons. Arrows indicate population means.

**Figure 2:**
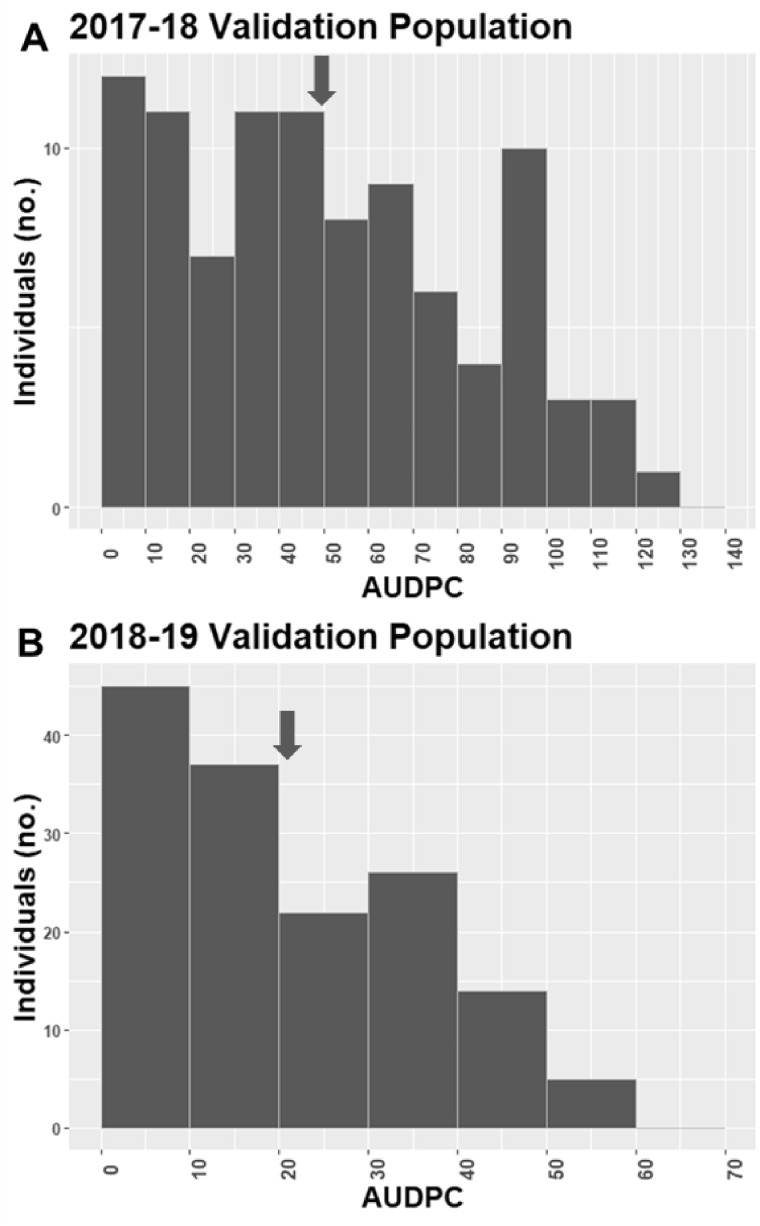
Distributions of AUDPC values for two QTLs validation populations tested over two field seasons. Arrows indicate population means.

**Figure 3:**
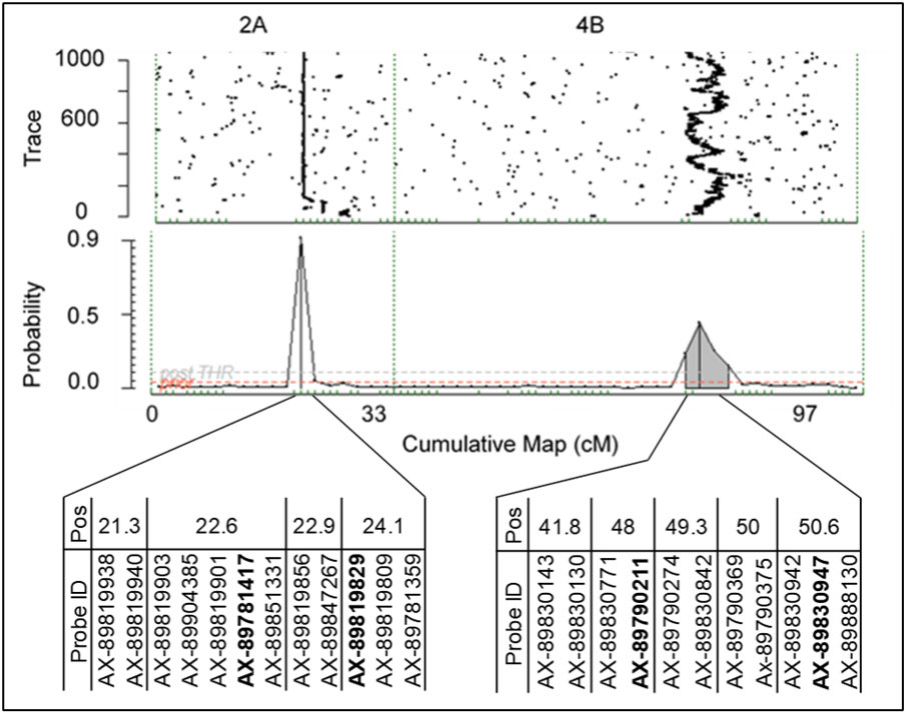
Trace plots and posterior probability graphs from Visual FlexQTL™ outputs showing the position of two QTL conferring resistance to plant collapse caused by *Macrophomina phaseolina*. SNP probes mapped to these locations on LGs 2A and 4B are shown. No other QTL with significant probabilities were observed. SNP probes in bold were used for haplotyping.

**Figure 4:**
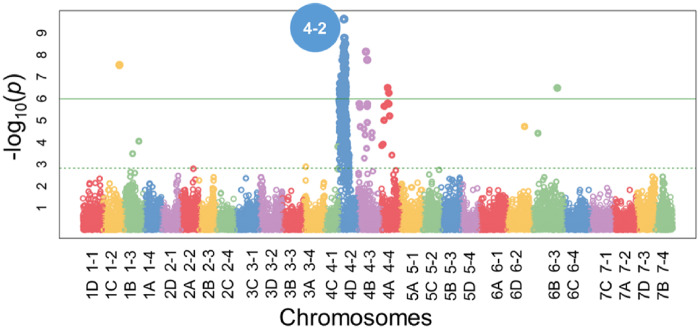
Manhattan plot from GWAS of the 2018–2019 discovery population derived from FVC 11–58. Chr4-2 corresponds to LG 4D. Chromosomes are named as per Edger *et al.* (2019) and Hardigan *et al.* (2020).

Wide variability in plant collapse due to *M. phaseolina* infection was observed in all populations tested. The AUDPC values in the discovery populations ranged from 0 to 90 in 2016–2017, 0–130 in 2017–2018, and 0–70 in 2018–2019. In all 3populations/years, both the resistant and susceptible extremes of the range were well-populated (Figure 1). In the validation populations, the ranges were similar with 0–130 in 2017–2018 and 0–60 in 2018–2019, but the distributions were weighted more heavily toward lower AUDPC values (resistance) (Figure 2). Two large-effect QTLs were detected with decisive evidence in UF breeding germplasm in both years (Figure 3). The first was located on LG 2A with an interval spanning 20.4to 24.9 cM (*FaRMp1*). The second was located on LG 4B spanning 41.1 cM to 61.2 cM (*FaRMp2*). LG 2A corresponds to chromosome (Chr) 2-2 of the “Camarosa” reference genome (Edger *et al.* 2019), and LG 4B corresponds to Chr4-3 (Hardigan *et al.* 2020). Naming of the two QTL followed the convention *Fragaria* resistance to *M. phaseolina* locus/gene *1*&*2*. For the discovery populations in 2018–2019 derived from FVC 11–58, a GWAS and a FlexQTL analysis were performed, in each of which a strong peak was observed on Chr 4-2 within the interval 7.0–8.0 Mb (Figure 4; Supplementary Figure 4). Chr4-2 corresponds to LG 4D of the genetic map (Edger *et al.* 2019; Hardigan *et al.* 2020). Therefore, FVC 11–58 carries a unique resistance locus, which we name *FaRMp3*.

The phenotypic effects of the two QTLs in UF germplasm jointly ranged from 13% to 36% of PVEaccording to FlexQTL™ outputs (Table 3). Narrow-sense heritabilities from the same analyses were in the range of 0.33 to 0.57 depending on the year and population. Thus, based on FlexQTL™ models, the two discovered QTL together accounted for approximately 1/3 to 2/3 of the heritable genetic variation for the trait. Broad-sense heritabilities from ASReml analyses, taking into account variability among clonal replicates, were 0.46 in 2016–2017 and 0.31 in 2017–2018, with substantial variance (0.19) apportioned to dominance in the second year. The estimate of PVEby *FaRMp3* in the FVC 11–58-derived crosses was 79%–82%, which is likely an overestimate when compared to a narrow-sense heritability from FlexQTL™ of 0.44 (Table 3). This overestimate appeared to be confirmed by single-marker ANOVAs in which the best SNPs from GWAS explained 44.3% (adjusted *R*^2^) of the phenotypic variability.

**Figure 5:**
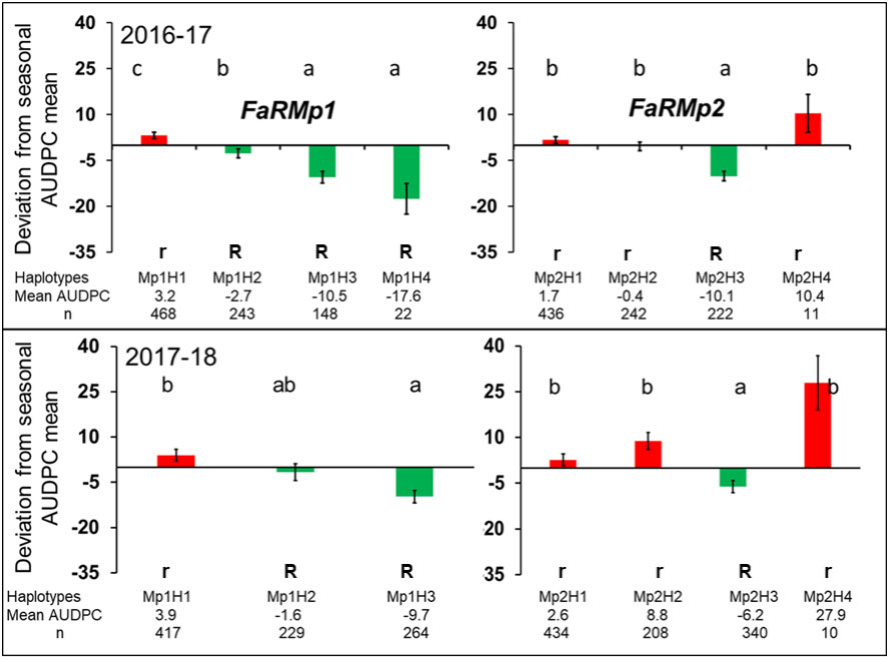
Deviations from AUDPC population means for individuals from two QTLs discovery populations tested in two different seasons by marker haplotypes at the *FaRMp1* and *FaRMp2* loci. Different letters represent statistically significant differences (*P < *0.05), as determined by Tukey’s multiple comparisons test. Haplotype sample sizes are shown at the bottom of each panel. Bars represent standard errors. R = resistant and r = susceptible QTL allele designations.

In the UF breeding populations, haplotyping was performed to characterize QTL alleles and their effects. Two SNP probes at each locus explained the haplotype variability in both of the QTL intervals, and all four possible marker haplotypes were present at both of the loci (Table 4). In both the discovery and validation populations, three haplotypes at each of the two loci predominated in both years (Supplementary Figure 5). Deviations of haplotypes from the AUDPC means of both discovery populations revealed both susceptibility- (positive AUDPC) and resistance-associated (negative AUDPC) haplotypes at both loci (Figure 5) that were consistent in direction of effect across both years/populations. Out of four haplotypes at *FaRMp1*, haplotype H1 had susceptible effect, and haplotypes H2, H3, and H4 had resistant effects. Haplotype H4 at *FaRMp1* was not present in the second-year discovery population. At *FaRMp2*, haplotypes H1, H2, and H4 had susceptible effects and H3 had a resistant effect. Diplotype (haplotype combination) effects in all discovery and validation populations were consistent with the direction of the haplotype effects (Supplementary Figure 6).

**Table 4:** Haplotypes at the *FaRMp1* and *FaRMp2* loci described by their phased SNP marker alleles

Haplotype	Allele 1	Allele 2	Haplotype	Allele 1	Allele 2
Mp1H1	T	A	Mp2H1	A	G
Mp1H2	T	C	Mp2H2	G	T
Mp1H3	G	C	Mp2H3	A	T
Mp1H4	G	A	Mp2H4	G	G
SNP Probe	AX-89781417	AX-89819829	SNP Probe	AX-89790211	AX-89830947
Position (cM)	22.9	24.1	Position (cM)	48.6	50.6

**Figure 6:**
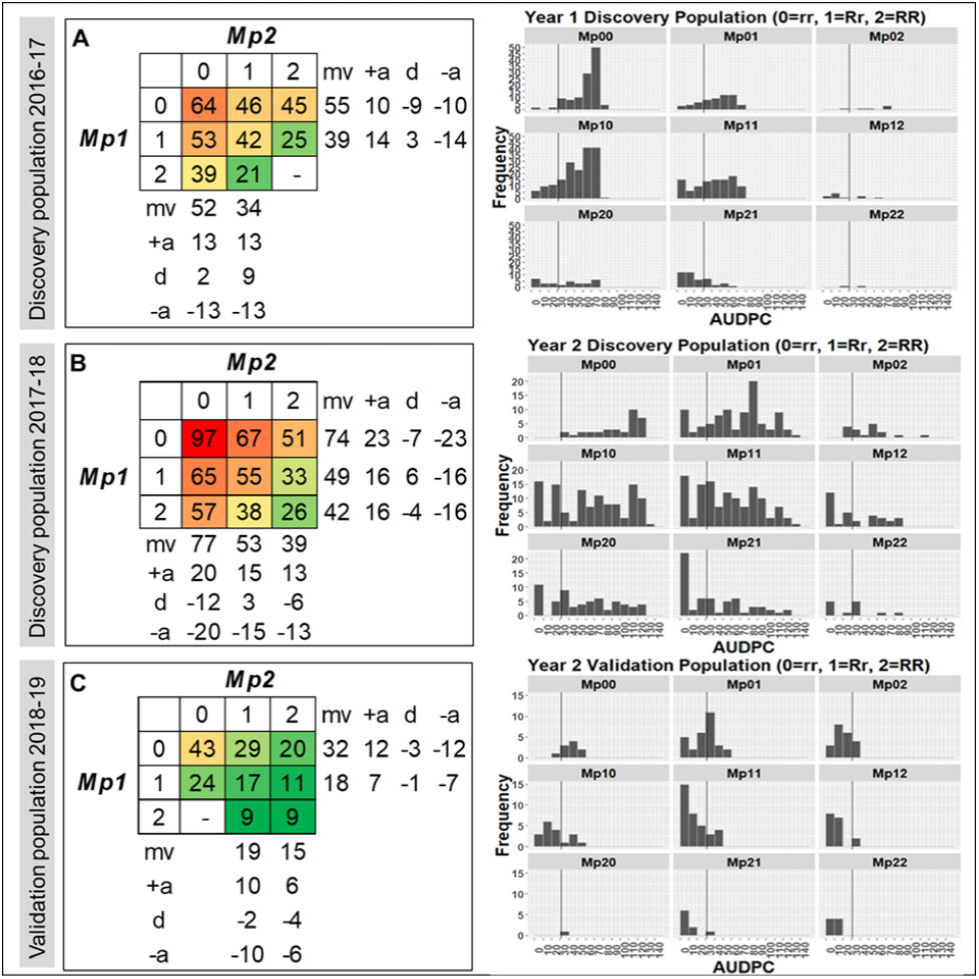
Phenotypic (AUDPC) effects of QTL genotype combinations at *FaRMp1* and *FaRMp2*. Genotypes are inferred from marker haplotypes in populations with sufficient sample size: (A) 2016–2017 discovery population, (B) 2017–2018 discovery population, and (C) 2018–2019 validation population. 0 = rr, 1 = Rr, 2 = RR, mv=mid value, a = additive genotypic value, and d=dominance. The left panel shows mean values of QTL genotype combinations overlaid with scaled color codes based on 3 years, and the right panel shows frequency distributions of AUDPC values for each QTL genotype combination. The line on each histogram represents an AUDPC of 31 for reference.

In order to further examine QTL allele effects within and across the two loci, haplotypes were assigned to resistant “R” or susceptible “r” alleles according to the direction of their effects (Figure 5). A summary of the mean AUDPC of the QTL genotype combinations is shown in Figure 6. The validation population in 2017–2018 was not included in this analysis due to small genotype sample sizes. There was some evidence for partial dominance at each locus when the other locus was homozygous for either the susceptible or resistant allele (Figure 6). However, the direction of dominance appeared to reverse for both loci when the other locus was heterozygous. This suggests some level of epistasis, but two-way ANOVAs did not support the presence of epistasis as there were no significant interactions (*P *≤* *0.05) between the two loci in any of the years/populations. There was an increase in resistance (decrease in AUDPC) when the number of resistant alleles increased from 0 to 4, regardless of the specific combination of alleles across the two loci. Generalizing across the three populations/years, an increase from 0 to 2 resistant alleles resulted in a 30%–60% reduction in AUDPC, while an increase from 0 to 4 resistant alleles resulted in a 73%–79% reduction in AUDPC (Figure 6).

## Discussion

To our knowledge this is the first report of loci contributing resistance to charcoal rot caused by *M. phaseolina* in strawberry. The discovery of *FaRMp1* on LG 2A and *FaRMp2* on LG 4B in cultivated germplasm was carried out in two different complex population sets, tested in two separate years. These populations were representative samples of the crosses made in the elite strawberry breeding population at UF. The two populations were highly related, even sharing multiple direct parents. Thus, alleles at both loci were well-replicated across populations and years. Furthermore, the effects of *FaRMp1* and *FaRMp2* on resistance were validated in two separate sets of cultivars and advanced selections across two seasons via the observation of consistent and significant marker haplotype (QTL allele) effects.

Average estimates of phenotypic effects of the two loci together from FlexQTL™ outputs were 27% in the first discovery population and 17% in the second discovery population consisting of UF breeding material. Comparing these estimates with heritability estimates from the same analyses (Table 3) suggests that one-third to two-third of the genetic variability for resistance was accounted for by the two loci. Such estimates are, of course, strongly influenced by allele frequencies. Becausethese elite populations were not made for the purpose of studying the inheritance of resistance, some crosses in both seasons did not segregate for either QTL. A more direct way to assess the potential utility of these loci in breeding is to measure the relative gains in resistance from selecting resistant alleles. This question was examined in the two discovery populations and the larger validation population in which the effects of QTL alleles and their interactions were quantified (Figure 6). Increasing from 0 to 2 resistance alleles (rrrr to RRrr, rrRR, or RrRr) would reduce the area under the disease progress curve by 30%–60%, while increasing from 0 to 4 resistance alleles (rrrr to RRRR) would reduce disease progress by 73%–79%. Thus, selecting for more resistant alleles at both loci should be an effective strategy for increasing resistance in the population and in future cultivars.

While we have demonstrated that loci for charcoal rot resistance exist within commercial germplasm, it is important to discover and characterize additional novel loci and alleles in order to increase the strength of resistance and its long-term durability. FVC 11–58 is a reconstituted *F. ×ananassa* of diverse parentage that was previously identified as resistant to charcoal rot (Zurn *et al.* 2020). This individual was crossed with susceptible selections from four different U.S. breeding programs and the four full-sib families screened for resistance, resulting in the discovery of *FaRMp3*. Becausethis population was genotyped with a next-generation array representing more complete chromosome coverage (Hardigan *et al.* 2020) and with SNP probes anchored to the “Camarosa” genome, a GWAS analysis was performed that confirmed the presence of a single locus on Chr4-2. The estimate of PVEby this locus was nearly 80%, which was not consistent with a narrow sense heritability estimate of 0.44. The reason for the lack of agreement between these two estimates was not clear but may relate to the high frequency of the resistant allele in the population. Regardless, the resistant effect of *FaRMp3* may be stronger than for the loci in commercial germplasm. Most of the FVC 11–58 progeny displayed greater resistance than commercial selections with a single dose of either *FaRMp1* or *FaRMp2.* The susceptible parents crossed to FVC 11–58 did not have resistant alleles at *FaRMp1* or *FaRMp2*. Therefore, we are not yet able to shed light on potential interactions among these loci and *FaRMp3*. Additional crosses have been made to examine the effects of pyramiding all three loci in future studies.

The *FaRMp2* (Chr 4-3) and *FaRMp3* (Chr 4-2) loci were located to different subgenomes of chromosome group four. Preliminary analysis of the two QTLs intervals suggests that *FaRMp2* and *FaRMp3* could reside in homoeologous regions, as they are both centered at physical positions of roughly 7–8 Mb on their respective subgenomes. The intervals will need to be narrowed considerably in order to further investigate the question of homoeology. We should note that a small number of significant markers were identified in the GWAS for the FVC 11–58 populations on Chr 4-3 and 4-4 in addition to 4-2 (Figure 4). As*FaRMp2* was not segregating in the FVC 11–58 populations, a possible explanation is that these markers were assigned to the wrong subgenomes and instead reside on 4-2.

In our field trials, plants of even the most resistant individuals eventually collapse with enough time under warm conditions. However, delaying plant collapse is still of great benefit to strawberry growers, allowing them to produce greater marketable yields. This is especially important since early-season yields are usually more profitable than late-season yields in most production regions of the world. Marker-assisted selection at *FaRMp1* and *FaRMp2* can be applied in the short term to reliably increase charcoal rot resistance in cultivars. Meanwhile, *FaRMp3* will be introgressed into cultivated germplasm. It will also be beneficial to characterize the genomic regions represented by these loci toward understanding the genes and causal polymorphisms involved. Given that genetic resistance is still the only effective post-fumigation control for charcoal rot of strawberry, the search for novel sources of resistance should continue in order to increase and diversify the genetic arsenal available to breeders.

## Data availability statement

A “Supplementary Figures” file contains six supplementary figures cited in this article and their captions. All marker genotypes, phenotypes, and pedigrees are available in additional supplementary data files. File S6, “Whitaker_pedigree_data policy documentation” contains detailed descriptions of Files S1–S5. File S1 contains genotypic, pedigree, phenotypic, and genetic map data for the 2016–2017 QTL discovery population set. File S2 contains genotypic, pedigree, phenotypic, and genetic map data for the 2017–2018 QTL discovery population set. File S3 contains genotypic data, physical map, and phenotypic data used for discovery population 2018–2019 GWAS analysis. File S4 has genotypic and phenotypic data used for the validation population 2017–2018. File S5 has genotypic and phenotypic data used for the validation population 2018–2019. Supplementary Material available at figshare: https://doi.org/10.25387/g3.13003700.
